# Anti-inflammatory effects of *Bifidobacterium infantis* M-63 during the early postnatal period in term infants

**DOI:** 10.1038/s41390-025-04263-y

**Published:** 2025-07-18

**Authors:** Chendong Xu, Toshitaka Odamaki, Akari Hiraku, Setsuko Nakata, Satoshi Arai, Noriyuki Iwabuchi, Miyuki Tanaka, Takahisa Tsuno, Masahiko Nakamura

**Affiliations:** 1 Innovative Research Institute, R&D Division, Morinaga Milk Industry Co., Ltd., Zama, Kanagawa Japan; 2 Department of Pediatrics, Matsumoto City Hospital, Matsumoto, Nagano Japan; 3 Matsuoka Pediatric Clinic, Matsumoto, Nagano Japan; 4 Department of Neurosurgery, Matsumoto City Hospital, Matsumoto, Nagano Japan

## Abstract

**Background:**

The administration of *Bifidobacterium infantis* M-63 during the early postnatal period enhances the abundance of gut *Bifidobacterium*, but its potential effects are still unexplored. The present study aimed to evaluate the impact of *B. infantis* M-63 on immunity, inflammation, gut-derived metabolites, and gut microbiota composition-based enterotypes in healthy infants.

**Methods:**

Fecal samples were collected from 111 healthy infants randomly administered 1.0 × 10^9^ CFU of *B. infantis* M-63 or placebo daily from 7 d to 3 months of age. Gut microbial composition characterization using 16S rRNA sequencing and genus-level enterotype clustering was performed. Fecal cytokine, metabolite, short-chain fatty acid, calprotectin, and secretory immunoglobulin A (sIgA) levels were measured.

**Results:**

Administering *Bifidobacterium infantis* M-63 significantly increased gut *Bifidobacterium*, whereas Enterobacteriaceae abundance and proinflammatory cytokine levels decreased. Six enterotypes were identified among the gut microbiota. In *Bifidobacterium-*dominant enterotypes, there was a significant increase in acetic acid and tryptophan metabolite levels, and a slight increase in sIgA levels. In contrast, levels of calprotectin and inflammatory cytokines were significantly reduced compared to those in the non-*Bifidobacterium* enterotypes.

**Conclusions:**

*Bifidobacterium*-dominant enterotypes, established in the gut after administration of *B. infantis* M-63, were strongly associated with anti-inflammatory effects in healthy infants.

**Impact:**

- This is the first study to demonstrate an anti-inflammatory effect in healthy full-term infants supplemented with *Bifidobacterium infantis* M-63 alone.
- *Bifidobacterium*-dominant enterotypes were associated with reduced levels of inflammatory cytokines and calprotectin, and increased production of beneficial tryptophan metabolites, such as Indole-3-lactic acid (ILA).
- This study provides evidence that supplementation with *B. infantis* M-63 in infants may significantly reduce inflammation during the critical early postnatal period.

## Introduction

The gut microbiota significantly influences infant health; early childhood development is pivotal in establishing a robust and diverse microbiome, with potentially long-term health implications.^1,2^ Various factors impact infant gut microbiota, including delivery mode, antibiotic exposure, feeding type, host genetics, and maternal health.^3^ Disturbances in the infant gut microbiota, termed gut dysbiosis, have been linked to inflammatory conditions, such as allergies or asthma,^4,5^ necrotizing enterocolitis,^6^ and celiac disease.^7^ Moreover, infant gut dysbiosis may increase the risk of type 1 diabetes^8^ and obesity^9^ later in life. Thus, the gut microbial composition is relevant to infant and adult health.

Gut microbiota disruptions in early life are associated with an immature immune system and can induce enterocolitis in infants.^10,11^ Gut dysbiosis elevates calprotectin levels, an inflammatory marker in the gut.^12,13^ Chronic inflammation has also been linked to the production of cytokines, which regulate host responses to infection, inflammation, and trauma.^14–16^ Inflammatory cytokines produced during inflammatory responses, such as tumor necrosis factor-α (TNF-α), interferon-γ (IFN-γ), and interleukin (IL)-1β, disrupt the intestinal adhesion barrier and further increase its permeability, which can exacerbate the inflammatory process.^17,18^ Cytokine production and tight junction permeability in the early postnatal period are associated with asthma and Crohn’s disease development in adults.^19,20^ Therefore, maintaining an appropriate cytokine balance in infancy is also important for adult health.

Bifidobacteria in the infant gut microbiota and breast milk intake play important roles in the early postnatal period.^2^ In particular, the mechanisms by which the gut microbiota affect host physiology have received considerable attention. Short-chain fatty acids (SCFAs), such as acetic acid, mediate this symbiotic relationship.^21^ The aromatic hydrocarbon receptor (AhR), a ligand-activated transcription factor widely expressed in cells, also acts as an environmental sensor that integrates immune responses in health and disease.^22,23^ Furthermore, the metabolites produced by gut bacteria, particularly bifidobacteria, are associated with anti-inflammatory activity. The tryptophan metabolites gut bacteria produce protect gut barrier function and suppress inflammatory responses. The loss of this tryptophan-converting ability leads to exacerbated inflammation and reduced AhR activation capacity in patients with inflammatory bowel disease.^24,25^ In addition, aromatic lactic acids from *Bifidobacterium* species may affect immune function in infants.^26^

*Bifidobacterium infantis* exhibits a remarkable adaptation to the gut of breastfed infants, with a high capacity to utilize human milk oligosaccharides (HMOs),^27^ which promote infant health and overall development.^28,29^ This phenomenon may result from the co-evolution of host and *B. infantis* via HMOs, which induce intestinal colonization during infancy.^30^ This colonization negatively correlates with Proteobacteria, which has adverse health effects.^31,32^ In addition, the absence of *B. infantis* is strongly associated with reduced vaccine responsiveness in infancy.^33^ *Bifidobacterium infantis* M-63 is a characteristic strain that prefers high availability of HMOs^34^ and exhibits lysozyme resistance in breast milk.^35^ It contributes to infant health, including regulating defecation frequency,^36^ improving abdominal symptoms,^37^ and reducing atopic dermatitis.^38^ However, its effects on inflammation remain unclear.

The concept of enterotypes, in which the gut microbiota is analyzed in clusters, has been recently proposed.^39^ Each enterotype that can be classified even in early postnatal infants represents a unique pattern of gut microbiota and is influenced by factors such as diet, geographical distance, lifestyle, and environment.^40,41^ Enterotypes based on gut microbiota composition are associated with SCFA profiles,^42^ and the maturation of microbiota enterotypes in the early postnatal period affects future health.^43^ The developmental transition of the gut microbiota during the first 2 years of life can be applied to health data.^44^ However, changes in gut microbiota enterotypes and individual-specific transitions during probiotic interventions remain under-explored.

In this study, we aimed to investigate the effects of *B. infantis* M-63 on gut microbiota composition, fecal cytokines, and metabolites in healthy-term infants. We further assessed the anti-inflammatory effect of gut microbiota composition, particularly the *Bifidobacterium*-dominant microbiota, on infant health.

## Methods

### Study design and participants

The present study is a single-center, placebo-controlled, double-blinded randomized trial, which is an additional analysis of a randomized clinical trial designed to evaluate the effect of *B. infantis* M-63 on healthy-term infants.^36^ Briefly, the study recruited healthy women who were to give birth to a healthy, full-term baby between October 2019 and August 2021. Eligible babies were healthy newborns born between 37 and 42 weeks. Exclusion criteria for mothers were a diagnosis of serious liver, kidney, cardiovascular, respiratory, endocrine, metabolic, or psychiatric disease, or planned use of other probiotics. Infant exclusion criteria were multiple birth, small for gestational age (SGA), large for gestational age (LGA), significant medical complications, exposure to antibiotics, and if deemed inappropriate by the investigator. Primary outcomes assessed relative abundance of *Bifidobacterium*, predominant proportion, and whether there was an increase in the number of copies in feces. Secondary outcomes included effects on gastrointestinal tract tolerance, child health (e.g., crying time, fever), fecal pH, SCFAs, sIgA, and calprotectin.

Healthy-term infants were randomized to receive either 1 billion colony-forming units of *B. infantis* M-63 (*B. infantis* M-63 group) daily or sterilized dextrin (placebo group) from ≤7 days to 3 months of age. Demographic information was collected for each participant and their parents. In addition, mothers kept daily records, including the number of times they fed their infants breast milk and formula. The study was sufficiently explained to the parents before enrollment, and written informed consent was obtained from the parents of all study participants. The study was conducted in accordance with the Declaration of Helsinki and approved by the Research Ethics Committee of Matsumoto City Hospital on September 27, 2019. The study was registered in the UMIN Clinical Trials Registry (UMIN000038351) in advance.

### Fecal sample collection

Two types of infant fecal samples were collected from the infants’ diapers by their parents using fecal collection tubes (Techno Suruga Laboratory Co., Ltd., Shizuoka, Japan): the first type were fresh samples before intake and at 1 month of age, and the second were those preserved with a guanidine thiocyanate solution before intake, 1 week after intervention, and at 1 month and 3 months of age. All the samples, collected at home, were transferred to the hospital and stored in −20 °C freezers. After being transported to the laboratory with dry ice, they were stored at −80 °C. Fresh fecal samples were used for SCFA, cytokine, and metabolite analyses, and fecal samples with preservative solution were used for 16S rRNA gene sequencing.

### Fecal DNA extraction and microbial, SCFA, and biomarker analysis

DNA extraction and microbial analysis were performed as described previously.^36^ The V3–V4 region of the bacterial 16S rRNA gene was amplified and sequenced on an Illumina MiSeq instrument (Illumina, San Diego, CA) for microbial analysis. Sequences aligned with GRCh38 and phiX reads were subsequently eliminated. The remaining sequences were analyzed using QIIME2 software version 2017.10. DADA2 was used to eliminate potential chimeric sequences and trim bases of the reads.^45^

Taxonomic classification was conducted using Greengenes 13.8 data, and alpha diversity was calculated with the QIIME2 software. Principal coordinate analysis (PCoA) and partitioning around medoid (PAM) clustering were conducted using R (version 4.3.0; R Foundation for Statistical Computing, Vienna, Austria). Enterotype clustering was performed at the genus level.^39^ Jensen–Shannon distance (JSD) and PAM clustering were applied for sample clustering, and the Calinski–Harabasz index was used to determine optimal enterotype clustering. Hierarchical clustering analysis was performed using the MeV suite version 4.9 (https://sourceforge.net/projects/mev-tm4/files/mev-tm4/MeV%204.9.0/), with distances calculated based on the Pearson correlation of enterotype transition data. Fecal SCFAs (acetic acid, propionic acid, and butyric acid) were determined using gas chromatography, whereas secretory immunoglobulin A (sIgA) and calprotectin were measured using the corresponding enzyme-linked immunosorbent assay kit as previously described.^36^

### Fecal cytokine analysis via multiplexed immunoassays

IL-1β and IL-8 levels were assessed using U-PLEX Biomarker Group 1 (human) Assays (Meso Scale Discovery, Rockville, MD). IFN-γ, IL-6, and TNF-α were measured with the S-PLEX Proinflammatory Panel 1 (human) Kit (Meso Scale Discovery). Additionally, IL-5 and IL-22 were quantified using the S-PLEX Human IL-5 and IL-22 Kits, respectively (Meso Scale Discovery), according to the manufacturer’s instructions.^46^

Briefly, 50–100 mg of fecal samples per participant were diluted 10-fold with a 100:1 mixed buffer of phosphate-buffered saline and proteinase inhibitor (Nacalai Tesque, Kyoto, Japan). Next, samples were agitated in a bead crusher (TAITEC, Saitama, Japan) with 5 mm stainless steel beads (QIAGEN, Valencia, CA) for three cycles of 2 min each. After centrifugation, the supernatant was collected, and each cytokine was measured using the respective kit. The standard and samples were assayed in duplicate using the Meso Scale Discovery (MSD) Quick Plex instrument and analyzed using the MSD software. Cytokine values were normalized to the fecal weight, and values below the detection limit were defined as zero.

### Metabolite extraction

To each stool sample, 10–40 mg, 200 μL of methanol solution was added. The sample was crushed at 2000 rpm for 30 s with 300 mg of zirconia beads, followed by centrifugation at 10,000 × *g* at 4 °C for 10 min, and the supernatant was collected. The supernatant volume was filtered through a Nanosep 3 Komega microconcentrator (Pall, Port Washington, NY) and centrifuged at 9100 × *g* at 4 °C for 120 min. The filtrate was collected and treated with a centrifugal evaporator for 30–60 min until dryness. All pretreated samples were stored at −20 °C before measurement.

### Quantification of fecal metabolite concentrations through LC-MS/MS

Fecal metabolites were determined using liquid chromatography–tandem mass spectrometry (LC-MS/MS) with a Vanquish high-performance liquid chromatograph coupled to TSQ-FORTIS (Thermo Fisher Scientific, Waltham, MA), as previously described.^47^ The analysis included 19 *B. infantis* M-63 and 23 placebo samples pre-ingestion, and 25 *B. infantis* M-63 and 16 placebo samples at 1 month. We measured indole-3-lactic acid (ILA), indole-3-aldehyde (IAld), indole-3-acetic acid (IAA), indole-3-propionic acid, 4-hydroxyphenyl-lactic acid (HPLA), 3-phenyl-lactic acid (PLA), tryptophan (Trp), tyrosine (Tyr), and phenylalanine (Phe) levels. A list of these compounds and their analytical conditions for selected reaction monitoring analysis is shown in Table S1.

LC-MS/MS quantification was performed using an XBridge® C8 column (4.6 × 150 mm, 3.5 μm) (Waters Corporation, Milford, MA). The mobile phases included water with 0.05% (v:v) formic acid (A) and methanol (B), with a flow rate of 0.2 mL/min. The gradient elution profile was as follows: (i) 2% B for 2 min, (ii) increase from 2% to 65% until 40 min, (iii) increase from 65% to 99% until 45 min, (iv) hold at 99% until 55 min, (vii) decrease from 99% to 2% until 60 min, and (viii) hold at 2% until 75 min. All chemical reagents used were of analytical grade.

### Statistical analysis

Differences in microbiota between groups at the phylum and genus levels were assessed using ALDEx2^48^; *q* < 0.05 was considered statistically significant. The Wilcoxon rank-sum test was used to compare the two groups, whereas Fisher’s exact test was used to analyze the distribution of infants within each enterotype or cluster. The Kruskal–Wallis test was used to confirm the presence of groups with the same distribution of cytokine levels, metabolites, and breastfeeding rate of infants in each enterotype, and Dunn’s multiple comparison test with Bonferroni correction was employed to identify differences within groups. Statistical analyses were conducted using R software (version 4.3.0) and SPSS software (version 28.0; IBM Corp., Armonk, NY); *p* < 0.05 was considered statistically significant.

## Results

### Study flow and characteristics

An overview of the clinical trial design and the flow diagram for this study is shown in Fig. S1. Of the 111 participants enrolled, 57 and 54 were randomized to the *B. infantis* M-63 and placebo groups, respectively. One participant in each group was excluded due to withdrawal of consent (before intervention) or low compliance, leaving 56 and 53 participants in the *B. infantis* M-63 and placebo groups, respectively. Detailed infant and maternal background information and safety results are available from a previous study.^36^ No significant differences in mode of delivery, birth circumstances, and other factors were observed between the groups, ensuring equal allocation to both groups. It should be noted that the results of the primary outcome and the secondary outcome have all been described in a previous paper.^36^

### Composition and diversity of the gut microbiome

**Fig. 1:**
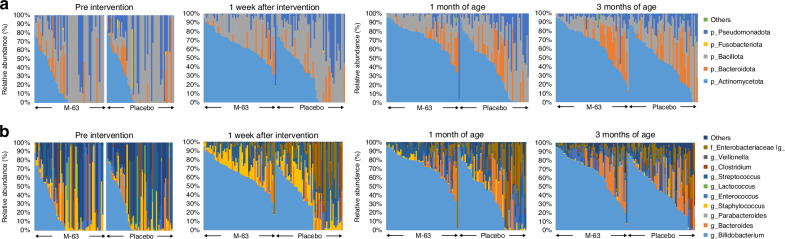
**Gut microbiota composition.** **a** The top five phyla between the *B. infantis* M-63 (*n* = 56) and placebo (*n* = 53) groups. The participants are organized in descending order based on the relative abundance of Actinomycetota at each time point. **b** The top 10 genera between the *B. infantis* M-63 and placebo groups. The participants are organized in descending order based on the relative abundance of *Bifidobacterium* at each time point. All other classes are collectively categorized and labeled as “others.” Due to unsuccessful DNA extraction at the pre-intervention stage, two samples from the *B. infantis* M-63 group and one from the placebo group were not included in the gut microbial analysis.

Taxonomic profiles of the gut microbiota among infants in both groups are shown in Fig. 1. The relative abundances of bacterial phyla and genera were compared between groups and over time using stacked vertical bars (Fig. 1a, b). Genera that significantly differed in relative abundance between groups included *Bifidobacterium* (1 week after intervention and at 1 month of age, *q* < 0.05) and Enterobacteriaceae of an unknown genus (1 month of age, *q* < 0.05). An increase in abundance of *Bifidobacterium* over time was observed with *B. infanits* M-63 administration during the intervention period, accompanied by a decrease in the abundance of Enterobacteriaceae (Table S2). Results for bacterial phyla and genera other than *Bifidobacterium* and Enterobacteriaceae are also shown in Table S2.

We also calculated the alpha diversity of the bacterial communities in both groups using several indices: Faith’s phylogenetic diversity (faith_PD), Shannon, Chao1, and the number of observed OTUs (observed_OTUs) (Table S3). The *B. infantis* M-63 group exhibited significantly lower diversity than the placebo group across all indices at 1 month of age (*p* < 0.05).

### Comparison of fecal metabolite and cytokine levels

**Fig. 2:**
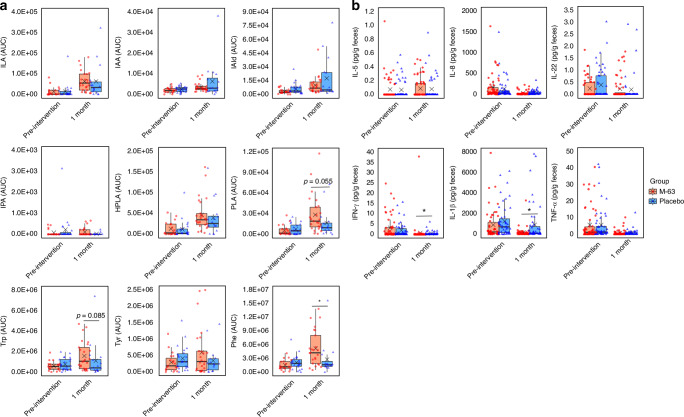
Fecal metabolites and cytokines between the two groups. Comparison of fecal metabolites (**a**) and cytokines (**b**). Each box plot represents the median, interquartile range, minimum, and maximum values. Intergroup differences at the pre-intervention point and 1 month of age were analyzed using the Wilcoxon rank-sum test. *p < 0.05 indicates statistical significance. AUC area under the curve.

The analysis of fecal metabolites revealed a significant increase in the amount of Phe in the *B. infantis* M-63 group (*p* < 0.05; Fig. 2a). Post-intervention, the amount of PLA (*p* = 0.055) and Trp (*p* = 0.085) tended to increase in the *B. infantis* M-63 group compared to the placebo group. However, these changes did not exhibit statistical significance. Additionally, no significant differences were observed between the two groups for tryptophan metabolites, such as ILA and IAld, or tyrosine metabolites, such as HPLA and PLA.

We further evaluated the inflammation status in the gut by measuring fecal cytokine levels (Fig. 2b). IL-2, IL-6, and IL-10 were excluded from the analysis due to their low detection rates (approximately 10%). The detection rate of measured cytokines showed no difference between the two groups at both time points (data not shown). However, the *B. infantis* M-63 group exhibited significantly lower concentrations of the proinflammatory cytokines IFN-γ and IL-1β than the placebo group (*p* < 0.05, each). No significant difference was observed in the levels of other cytokines at 1 month of age.

### Analysis of enterotypes and breastfeeding rates

**Fig. 3:**
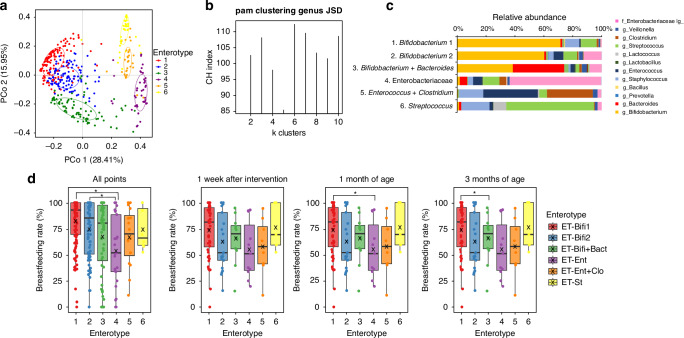
Characteristics of enterotypes and their breastfeeding rates. **a** PCoA is based on the JSD calculated from the fecal microbiota composition at the genus level. **b** PAM clustering with JSD, including the estimated suitability of the cluster number using the Calinski–Harabasz index. **c** The fecal microbiota composition in each enterotype. **d** The breastfeeding rate in each enterotype. The Kruskal–Wallis and Dunn–Bonferroni post-hoc tests were used to compare each enterotype group; *p < 0.05 indicates statistical significance. PCoA principal coordinate analysis, JSD Jensen–Shannon distance, PAM partitioning around medoid.

Fecal samples were collected from 109 individuals at four sampling sites, and 433 samples were used, as DNA could not be extracted from three fecal samples. Assessment of the evolution of the infant gut microbiota during intervention using PCoA revealed six distinct enterotypes (Fig. 3a, b). The composition of the gut microbiota of each enterotype is summarized in Fig. 3c. Enterotypes 1 and 2 were *Bifidobacterium*-dominant (ET-Bifi 1, ET-Bifi 2), whereas enterotype 3 had a balanced presence of *Bifidobacterium* and *Bacteroides* (ET-Bifi and Bact), indicative of a healthy infant gut microbiota. However, non-*Bifidobacterium*-dominant microbiota exhibited a significant shift toward facultative anaerobic bacteria. Specifically, enterotype 4 was Enterobacteriaceae-dominant (ET-Ent), enterotype 5 comprised *Enterococcus* and *Clostridium* (ET-Ent and Clo), and enterotype 6 was *Streptococcus*-dominant (Et-St). Table S4 shows the distribution of enterotypes between the two groups.

We further calculated breastfeeding rates for each enterotype (Fig. 3d). ET-Bifi 1 (Average, 82.3%) and ET-Bifi 2 (Average, 74.6%) had significantly higher breastfeeding rates than ET-Ent (Average, 52.7%) at all points (*p* < 0.05), with differences also observed between ET-Bifi 1 (Average, 85.4%) and ET-Ent (Average, 48.5%) at 1 month of age (*p* < 0.05). No differences in breastfeeding rates were observed between the two groups in each enterotype (data not shown).

### Fecal metabolite, cytokine, SCFA, and biomarker levels based on microbial enterotypes

**Fig. 4:**
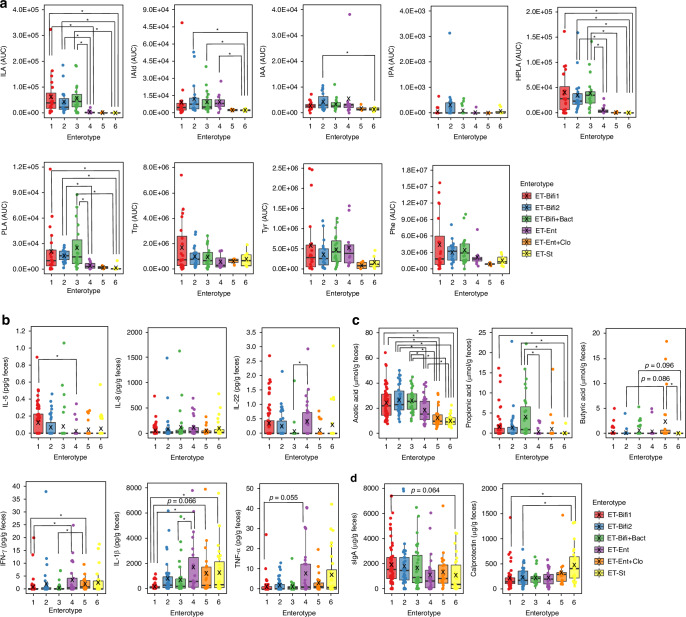
Biomarker profiles in each enterotype. **a** Fecal metabolites. **b** Fecal cytokines. **c** SCFAs. **d** sIgA and calprotectin. The Kruskal–Wallis test and Dunn–Bonferroni post-hoc tests were applied to compare each enterotype group; *p < 0.05 indicates statistical significance. AUC area under the curve, SCFA short-chain fatty acids, sIgA secretory immunoglobulin A.

We examined metabolite production patterns based on enterotypes (Fig. 4a). ILA, which showed no significant differences between groups, exhibited a predominant increase in ET-Bifi 1, ET-Bif 2, and ET-Bifi and Bact compared to non-*Bifidobacterium*-dominant enterotypes. This suggests a strong correlation between *Bifidobacterium* abundance and ILA production. Similarly, IAld and IAA levels increased significantly in *Bifidobacterium*-dominant enterotypes. Other aromatic lactic acids, such as HPLA and PLA, also demonstrated significantly higher levels in ET-Bifi 1, ET-Bif 2, and ET-Bifi and Bact (*p* < 0.05). In contrast, no differences in Trp, Tyr, and Phe levels were observed between the enterotypes.

We investigated potential variations in cytokine production based on enterotypes (Fig. 4b). IFN-γ levels in ET-Bifi 1 were significantly lower than those in ET-Ent and ET-Ent and Clo (*p* < 0.05). Similarly, IL-1β production in ET-Bifi 1 was significantly lower than in ET-Ent and Et-St (*p* < 0.05) and was lower than in ET-Ent and Clo (*p* = 0.066). IL-5 was significantly higher in ET-Bifi 1 than in ET-Ent (*p* < 0.05), whereas TNF-α exhibited a trend toward lower levels in ET-Bifi 1 than in ET-Ent (*p* = 0.055), although the differences were not significant. Significant differences in IL-22 production were observed between ET-Bifi and Bact and ET-Ent (*p* < 0.05). However, no difference in IL-8 production was observed among enterotypes.

*Bifidobacterium*-dominant microbiota such as ET-Bifi 1 and ET-Bifi and Bact (*p* < 0.05) mainly produced acetic acid and propionic acid, whereas butyric acid was predominantly produced by ET-Ent and Clo (*p* < 0.05) (Fig. 4c). Furthermore, sIgA production was higher in ET-Bifi 1 than in ET-St (*p* = 0.064). In contrast, calprotectin production was significantly higher in ET-St than in the *Bifidobacterium*-dominant type (*p* < 0.05) (Fig. 4d).

### Dynamic analysis based on gut microbiota changes during the first 3 months

**Fig. 5:**
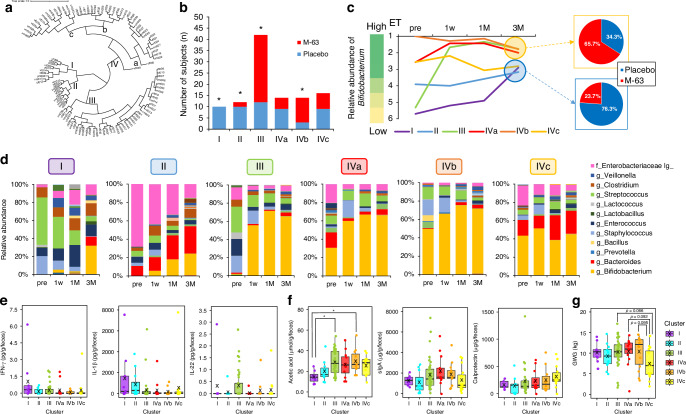
Grouping based on the dynamics of the gut microbiota during the intervention. **a** Cluster analysis relying on the dendrogram. **b** Distribution of participants in each cluster within the *B. infantis* M-63 and placebo groups (*p < 0.05 indicates statistical significance between the two groups, as determined using Fisher’s exact test). **c** Transition, indicating a change in enterotype, during the first 3 months. **d** The gut microbial composition of each cluster. **e** Fecal cytokines. **f** Acetic acid, sIgA, calprotectin. **g** GWG. The Kruskal–Wallis and Dunn–Bonferroni post-hoc tests were used to compare each cluster; *p < 0.05 indicates statistical significance. sIgA secretory immunoglobulin A, GWG gestational weight gain.

We performed hierarchical clustering analysis according to enterotypes to pattern and visualize the dynamics of the gut microbiota in each participant. We then classified the participants into six clusters (Fig. 5a). Significant differences were observed in the distribution of the number of enterotype participants in clusters I, II, III, and IVb (*p* < 0.05, as determined using Fisher’s exact test) (Fig. 5b). In addition, cluster III showed a rapid increase in *Bifidobacterium* abundance within the first week after intervention, whereas clusters I and II, characteristic of the placebo group, showed minimal or slow increases in *Bifidobacterium* abundance (Fig. 5c). At 3 months of age, clusters III, IVa, and IVb, evolving into ET-Bifi 1 or ET-Bifi 2, had a significantly higher proportion of *B. infantis* M-63 (65.7%) than the other clusters (23.7%) (Fig. 5c). Figure 5d shows the detailed microbial composition of each cluster from pre-intervention to 3 months of age.

Cytokine levels were analyzed for each cluster, and representative cytokine results are shown in Fig. 5e. IL-1β production was higher in clusters I and II, whereas IL-22 was higher in cluster III than in other clusters, but no statistical differences were observed. Acetic acid production was significantly higher in cluster III and cluster IVb than in cluster I (*p* < 0.05) (Fig. 5f). Although not statistically significant, sIgA levels were low, and calprotectin levels were high in cluster IVc. In addition, maternal gestational weight gain (GWG) tended to be lower than that of mothers of infants in other clusters (Fig. 5f, g). Figure S2 shows the changes in breastfeeding rate for each cluster over time.

## Discussion

The gut microbiota exerts physiological effects on the host,^49^ varying widely among individuals. Establishing balanced gut microbiota in the early postnatal period is critical for healthy development.^2,50^ In this study, we utilized fecal samples from a previous probiotic intervention study in neonates^36^ to evaluate the effect of *B. infanits* M-63 on fecal cytokine and metabolite production. We further explored the relationship between gut microbiota composition and fecal cytokine and metabolite production based on enterotypes and characterized the *Bifidobacterium*-dominant gut microbiota. We observed decreased inflammation-related biomarkers and increased metabolites with anti-inflammatory properties within a *Bifidobacterium*-rich environment. These findings elucidate the relationship between the gut microbiota and anti-inflammatory properties during the early postnatal period.

Our research indicates that early postnatal intake of *B. infantis* M-63 contributes to establishing a healthy gut environment by significantly increasing the abundance of *Bifidobacterium* and decreasing that of Enterobacteriaceae associated with various gut-related diseases and dysbiosis (Table S2).^51^ *B. infantis* M-63 intake led to a rapid increase in gut *Bifidobacterium* and a decrease in alpha diversity. The lower diversity in the probiotic group is associated with a rapid increase in *Bifidobacterium* abundance, consistent with a previous observation that breastfed infants have higher *Bifidobacterium* levels and thus lower diversity than formula-fed infants.^52^ However, unlike previous studies involving bifidobacteria in neonates,^53^ we did not observe an increase in tryptophan metabolites (ILA, IAA, IAld) in our comparison of gut metabolites. This difference is possibly due to the intervention subjects being healthy-term infants, with a general tendency to increase bifidobacteria naturally, and the limited sample size. Therefore, it was necessary to focus on the gut microbiota classification for further analysis.

Leveraging gut microbiota data from 433 samples collected from both the placebo and probiotic groups, spanning the neonatal period to 3 months of age, we conducted an enterotype classification. This analysis showed that the gut microbiota could be categorized into six types, significantly influenced by the breastfeeding rate during the intervention period (Fig. 3). Previous studies have characterized the gut microbial profile during the first month of life by the abundance of Bifidobacteriaceae, Enterobacteriaceae, and Staphylococcaceae.^41^ In contrast, the first 2 years of life exhibit a predominance of Bifidobacteriales, Enterobacterales, and Clostridiales.^42^ Our results are consistent with these findings and accurately reflect the gut microbiota of infants during the neonatal period and early infancy. Furthermore, no previous studies using double-blind clinical trial data have classified the infant gut microbiota into enterotypes and analyzed gut cytokines and metabolites in detail. Our study is unique in that it investigates the association between gut microbiota and the production of cytokines and metabolites in neonates up to 3 months of age. The data covering a period during which infants are exclusively breastfed or formula-fed provides valuable insights into how enterotypes based on gut microbiota are associated with cytokine and metabolite production and their impact on the host.

Our enterotype analysis demonstrates that the abundance of *Bifidoabcgerium* in the gut induces the production of tryptophan metabolites (ILA, IAld, IAA, HPLA, and PLA). These tryptophan metabolites contribute to improving intestinal barrier function and immune response, as well as immunomodulation by maintaining the intestinal barrier and fighting bacterial infections.^26,54,55^ SCFA, which increases in *Bifidobacterium*-dominant enterotypes, is closely associated with delaying the progression of various diseases, including those of the immune system, where it exhibits allergy suppression and anti-inflammatory properties.^56,57^ Furthermore, *Bifidobacterium*-dominant gut microbiota is associated with increased sIgA levels and decreased fecal calprotectin levels (Fig. 4d), which may exert anti-inflammatory and anti-infective effects.^58^ Although this study shows an association between enterotypes and biological indicators, the limitations of enterotype bias are unavoidable. This is because the participants were full-term infants, and by 3 months of age, even infants in the placebo group showed a spontaneous increase in *Bifidobacterium*. This may cause a bias in the number of enterotypes, resulting in fewer non-*Bifidobacterium*-dominant enterotypes. Our analysis of enterotypes provides the characteristics of the gut microbiota, particularly the *Bifidobacterium* dominance, in relation to specific bacterial metabolites.

Furthermore, focusing on the differences in the formation process of gut microbiota, we analyzed the impact of the early formation of a *Bifidobacterium*-dominated gut microbiota by clustering the process of gut microbiota change. Comparison of gut microbiota migration patterns between the *B. infantis* M-63 and placebo groups revealed that by 3 months, they had settled on the two main enterotypes, ET-Bifi 2 or ET-Bifi and Bact, indicating an important role for *Bifidobacterium* and *Bacteroides* in the early gut.^44^ No statistically significant differences were found in the metabolites and cytokine production; only IL-22 was observed more frequently in cluster III, suggesting that ILA from bifidobacteria may promote IL-22 transcription via the AhR.^55^ Additionally, cluster analysis showed that GWG was associated with the infant gut microbiota development pattern. Adequate GWG is important for maternal health and normal fetal development, while inadequate GWG is associated with an increased risk of low birth weight, preterm birth, and cesarean section.^59^ Infants born to mothers with insufficient GWG showed little increase in *Bifidobacterium*, lower fecal sIgA levels, and elevated calprotectin production (Fig. 5c, d, g). By tracing the transition of the gut microbiota in the early postnatal period, it was possible to identify the characteristics of each group, the metabolites that change with the transition, and the associated factors.

The transition of the gut microbiota in the early postnatal period is influenced not only by probiotic interventions but also by the breastfeeding rate.

The breastfeeding rate was lower in *B. infantis* M-63 groups in clusters II and IVc than in III, IVa, and IVb (Fig. S2). Importantly, *Bifidobacterium infantis* is genetically characterized by its ability to consume HMOs and use them efficiently for growth.^60,61^ The slow increase in *Bifidobacterium* abundance in some infants in the *B. infantis* M-63 group may be due to low breastfeeding rates and lack of HMO use. In other words, different breastfeeding rates also contribute to the transitions in each cluster. These observations suggest that breastfeeding, together with the intake of bifidobacteria as probiotics, is beneficial for the early establishment of a *Bifidobacterium*-dominant gut microbiota, as the formation of the gut microbiota varies greatly between individuals.

This study has some limitations. The number of non-*Bifidobacterium*-dominant enterotypes was small because the study was conducted in healthy-term infants. In addition, observing changes in the gut microbiota in the *B. infantis* M-63 group over a longer period, such as one or 2 years, may provide more insight into its effects and the relationship between altered biomarkers and infant health. Furthermore, the establishment of the gut microbiota during infancy is influenced not only by bifidobacterial interventions but also by breast milk. However, we did not collect breast milk samples, so the effect of breast milk HMO components on the infant gut microbiota could not be evaluated. In the future, large, multi-center clinical trials may help to better understand the influence of *B. infantis* M-63 on gut microbiota, biomarkers, and infant health.

In conclusion, our findings demonstrate that gut microbiota enterotypes in neonates and early infancy are strongly associated with cytokine and metabolite production. Specifically, a *Bifidobacterium*-dominant microbiota resulted in a decreased level of inflammatory cytokines and fecal calprotectin, along with an increase in the production of beneficial metabolites that alleviate local and systemic inflammation, such as ILA. *B. infantis* M-63 maintains a healthy gut environment by promoting *Bifidobacterium* abundance and reducing inflammatory Enterobacteriaceae species. Thus, supplementation with *Bifidobacterium* in the early postnatal period could establish a healthy gut microbiota during crucial immune development, potentially providing long-term health benefits to infants.

## Supplementary information

Supplemental material

## Data Availability

Data sets generated and analyzed during the current study are available from the corresponding author upon reasonable request.
