# Supplementary material for: Anti-inflammatory effects of Bifidobacterium infantis M-63 during the early postnatal period in term infants
Source: Pediatr Res. 2025 Jul 18;99(5):1842–50. doi: 10.1038/s41390-025-04263-y (PMC13221305; doi:10.1038/s41390-025-04263-y)
Supplement: Supplementary file 1 — Supplemental material [file 41390_2025_4263_MOESM1_ESM.pdf]

Table S1. List of the compounds and their analytical conditions of SRM (Selected reaction monitoring) analysis.

Compound	Polarity	Precursor (m/z)	Product (m/z)	Collision Energy (V)	CAS RN	Purchasing source
Tyr	Positive	182.088	165.054	8.79	60-18-4	Merck, Japan, Tokyo, Japan
Phe	Positive	166.088	120.054	11.66	63-91-2	Merck, Japan, Tokyo, Japan
Trp	Positive	205.125	188.125	8.92	73-22-3	Merck, Japan, Tokyo, Japan
HPLA	Negative	181.000	163.054	11.70	306-23-0	Merck, Japan, Tokyo, Japan
ILA	Positive	206.088	118.054	20.33	832-97-3	Merck, Japan, Tokyo, Japan
IAA	Positive	176.088	130.054	13.55	87-51-4	Merck, Japan, Tokyo, Japan
PLA	Negative	165.000	147.125	10.81	20312-36-1	Merck, Japan, Tokyo, Japan
IAld	Positive	146.088	118.054	13.76	487-89-8	Merck, Japan, Tokyo, Japan
IPA	Positive	190.212	130.125	14.31	830-96-6	Merck, Japan, Tokyo, Japan

Table S2. Relative abundance of infant fecal microbiota measured by 16s rRNA amplicon sequencing.

		B. infantis M-63	Placebo
		Median (IQR) %	
Pre intervention			
(M-63 n=54, Placebo n=52)	**Phylum**		
	Actinomycetota	1.41 (0.12-34.4)	0.15 (0.00-34.8)
	Bacillota	43.7 (18.5-98.8)	34.4 (14.4-99.7)
	Pseudomonadota	0.34 (0.00-22.2)	6.01 (0.00-33.7)
	**Genus**		
	Bifidobacterium	0.19 (0.06-33.2)	0.13 (0.00-39.0)
	*Enterobacteriaceae|g_*	0.07 (0.00-19.8)	0.30 (0.00-27.7)
	Enterococcus	0.00 (0.00-3.27)	0.00 (0.00-6.91)
	Lactococcus	0.14 (0.00-0.48)	0.19 (0.00-0.74)
	Staphylococcus	3.14 (0.41-14.6)	1.55 (0.38-7.12)
	Streptococcus	1.18 (0.14-20.3)	5.85 (0.18-47.5)
	Veillonella	0.00 (0.00-0.00)	0.00 (0.00-0.00)
1 week after intervention			
(M-63 n=56, Placebo n=53)	**Phylum**		
	Actinomycetota	65.0 (53.4-75.0)	28.4 (0.19-52.5) *
	Bacillota	26.2 (12.5-36.8)	43.5 (28.3-65.9) *
	Pseudomonadota	0.51 (0.00-4.85)	7.43 (0.05-28.9) *
	**Genus**		
	Bifidobacterium	65.0 (52.9-75.0)	28.4 (0.08-52.5) **

	*Enterobacteriaceae|g_*	0.12 (0.00-3.73)	4.53 (0.00-19.1)
	Enterococcus	0.00 (0.00-0.78)	0.08 (0.00-5.61)
	Lactococcus	0.00 (0.00-0.00)	0.00 (0.00-0.00)
	Staphylococcus	9.14 (3.46-19.3)	9.91 (2.99-23.6)
	Streptococcus	6.34 (0.99-13.0)	8.38 (2.52-21.9)
	Veillonella	0.00 (0.00-0.00)	0.00 (0.00-0.00)
1 month of age			
(M-63 n=56, Placebo n=53)	**Phylum**		
	Actinomycetota	77.5 (64.0-82.5)	43.5 (2.15-56.5) **
	Bacillota	16.4 (10.9-21.1)	32.2 (17.9-45.2)
	Pseudomonadota	0.69 (0.00-3.28)	8.22 (1.42-20.6) **
	**Genus**		
	Bifidobacterium	77.5 (63.3-82.3)	43.5 (0.24-56.4) **
	*Enterobacteriaceae|g_*	0.18 (0.00-2.74)	3.82 (0.12-16.6) *
	Enterococcus	0.00 (0.00-2.30)	0.27 (0.00-3.28)
	Lactococcus	0.00 (0.00-0.00)	0.00 (0.00-0.00)
	Staphylococcus	0.76 (0.22-1.77)	0.97 (0.12-3.10)
	Streptococcus	7.65 (2.73-12.4)	10.9 (1.71-18.5)
	Veillonella	0.00 (0.00-1.33)	0.32 (0.00-3.93)
3 months of age			
(M-63 n=56, Placebo n=53)	**Phylum**		
	Actinomycetota	71.7 (48.3-80.8)	46.8 (23.4-62.9) †

Bacillota	15.2 (8.95-22.3)	23.5 (13.4-40.2)
Pseudomonadota	2.97 (1.32-6.30)	7.67 (2.14-16.7)
**Genus**		
Bifidobacterium	71.4 (46.6-80.6)	44.2 (23.4-62.6) †
*Enterobacteriaceae|g_*	2.69 (0.72-4.51)	6.73 (1.26-16.5)
Enterococcus	1.33 (0.00-10.4)	1.34 (0.25-3.59)
Lactococcus	0.00 (0.00-0.00)	0.00 (0.00-0.00)
Staphylococcus	0.12 (0.00-0.38)	0.13 (0.00-0.55)
Streptococcus	1.72 (0.61-3.53)	2.61 (0.82-8.46)
Veillonella	1.95 (0.00-5.18)	1.18 (0.00-3.36)

Data are expressed as the median (IQRs) of the taxa with a median relative abundance of 0.1% in at least one group.

Intergroup differencens were analyzed by ALDEx2. †q＜0.1, *q＜0.05, **q＜0.01

IQR: Interquartile Range

Table S3. Alpha diversity of fecal microbiota in *B. infantis* M-63 and placebo groups.

		B. infantis M-63	Placebo
Faith_PD	Pre intervention	3.75 (3.61-4.24)	3.83 (3.52-4.24)
	1 week after intervention	3.79 (3.54-4.39)	3.98 (3.69-4.49)
	1 month of age	4.13 (3.73-4.69)	4.68 (4.24-5.06) *
	3 months of age	4.49 (4.08-5.44)	4.79 (4.46-5.27)
Shannon	Pre intervention	1.63 (1.20-2.23)	1.70 (1.17-2.28)
	1 week after intervention	1.66 (1.31-2.12)	2.31 (1.70-2.60) *
	1 month of age	1.37 (0.92-2.11)	2.35 (1.96-2.80) *
	3 months of age	1.80 (1.24-2.48)	2.64 (2.40-3.07) *
Chao1	Pre intervention	10.0 (7.25-14.0)	10.0 (8.00-13.0)
	1 week after intervention	10.0 (7.75-15.0)	12.0 (9.00-15.0)
	1 month of age	12.0 (8.00-17.0)	16.0 (14.0-19.0) *
	3 months of age	18.0 (11.0-23.3)	19.0 (16.0-23.0)
Observerd_OTUs	Pre intervention	9.00 (7.25-14.0)	10.0 (8.00-12.3)
	1 week after intervention	10.0 (7.00-15.0)	12.0 (9.00-15.0)
	1 month of age	12.0 (8.00-17.0)	16.0 (14.0-19.0) *
	3 months of age	18.0 (11.0-23.0)	19.0 (16.0-22.0)

Data are presented as the medians (interquartile ranges).

The Wilcoxon rank sum test was used to analyze differences between the two groups.

* $p < 0.05$

Table S4. Distribution of gut microbiota enterotypes by number of subjects

	Group	ET-Bifi1 n, (%)	ET-Bifi2 n, (%)	ET-Bifi and Bact n, (%)	ET-Ent n, (%)	ET-Ent and Clo n, (%)	ET-St n, (%)
Pre intervention							
	M-63 (n=54)	10 (18.5)	5 (9.3)	7 (13.0)	9 (16.7)	13 (24.1)	10 (18.5)
	Placebo (n=52)	3 (5.8)	11 (21.2)	7 (13.5)	10 (19.2)	4 (7.7)	17 (32.7)
	Total (n=106)	13 (12.3)	16 (15.1)	14 (13.2)	19 (17.9)	17 (16.0)	27 (25.5)
1 week after intervention							
	M-63 (n=56)	38 (67.9)	11 (19.6)	6 (10.7)	1 (1.8)	0 (0.0)	0 (0.0)
	Placebo (n=53)	14 (26.4)	10 (18.9)	6 (11.3)	10 (18.9)	8 (15.1)	5 (9.4)
	Total (n=109)	52 (47.7)	21 (19.3)	12 (11.0)	11 (10.1)	8 (7.3)	5 (4.6)
1 month of age							
	M-63 (n=56)	36 (64.3)	11 (19.6)	8 (14.3)	1 (1.8)	0 (0.0)	0 (0.0)
	Placebo (n=53)	12 (22.6)	13 (24.5)	11 (20.8)	9 (17.0)	6 (11.3)	2 (3.8)
	Total (n=109)	48 (44.0)	24 (22.0)	19 (17.4)	10 (9.2)	6 (5.5)	2 (1.8)
3 months of age							
	M-63 (n=56)	17 (30.4)	20 (35.7)	18 (32.1)	0 (0.0)	0 (0.0)	1 (1.8)
	Placebo (n=53)	7 (13.2)	25 (47.2)	15 (28.3)	4 (7.5)	2 (3.8)	0 (0.0)
	Total (n=109)	24 (22.0)	45 (41.3)	33 (30.3)	4 (3.7)	2 (1.8)	1 (0.9)

2 samples in the *B. infantis* M-63 group and 1 sample in the placebo group were excluded from the analysis due to unsuccessful DNA extraction at pre intervention point.

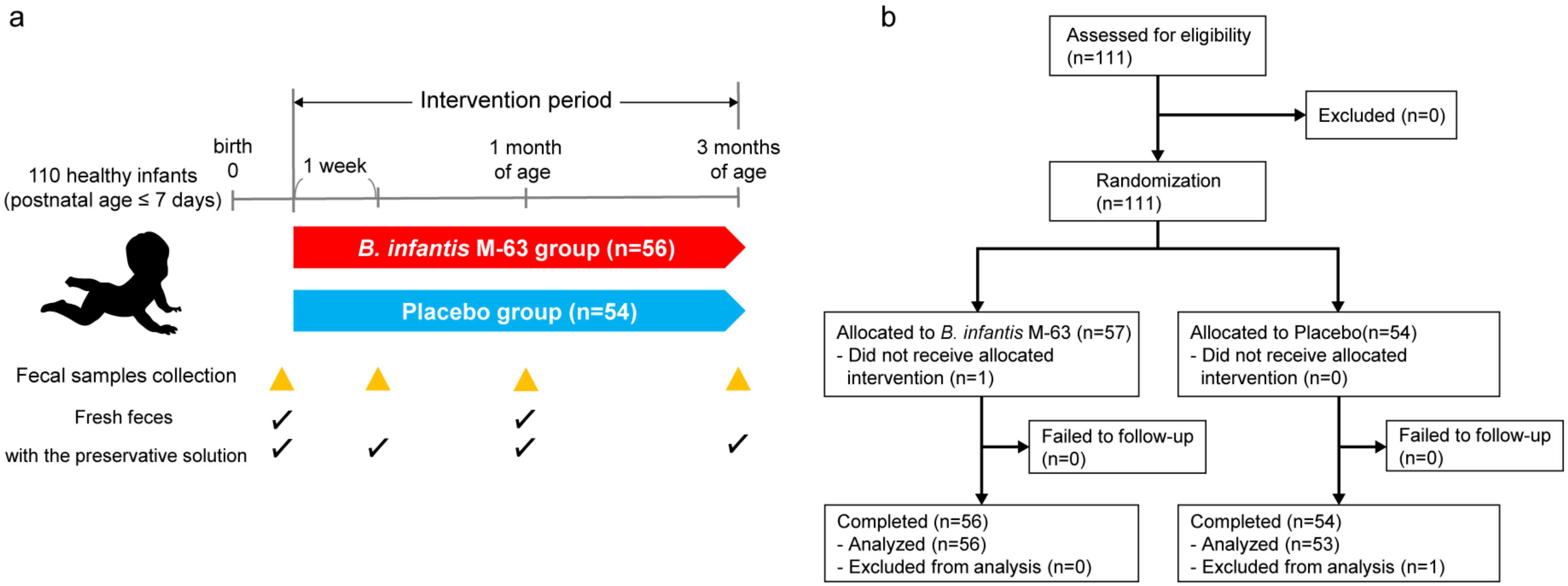

Figure S1. Clinical trial design. (a) Overview of the clinical trial design. (b) A CONSORT flowchart of participant recruitment and allocation to the study groups. One participant in the *B. infantis* M-63 group withdrew from the study by withdrawing consent before consumption of the test food, and one participant in the placebo group was excluded from the analysis due to low compliance. Participants began taking either *B. infantis* M-63 or a placebo within 7 d of birth.

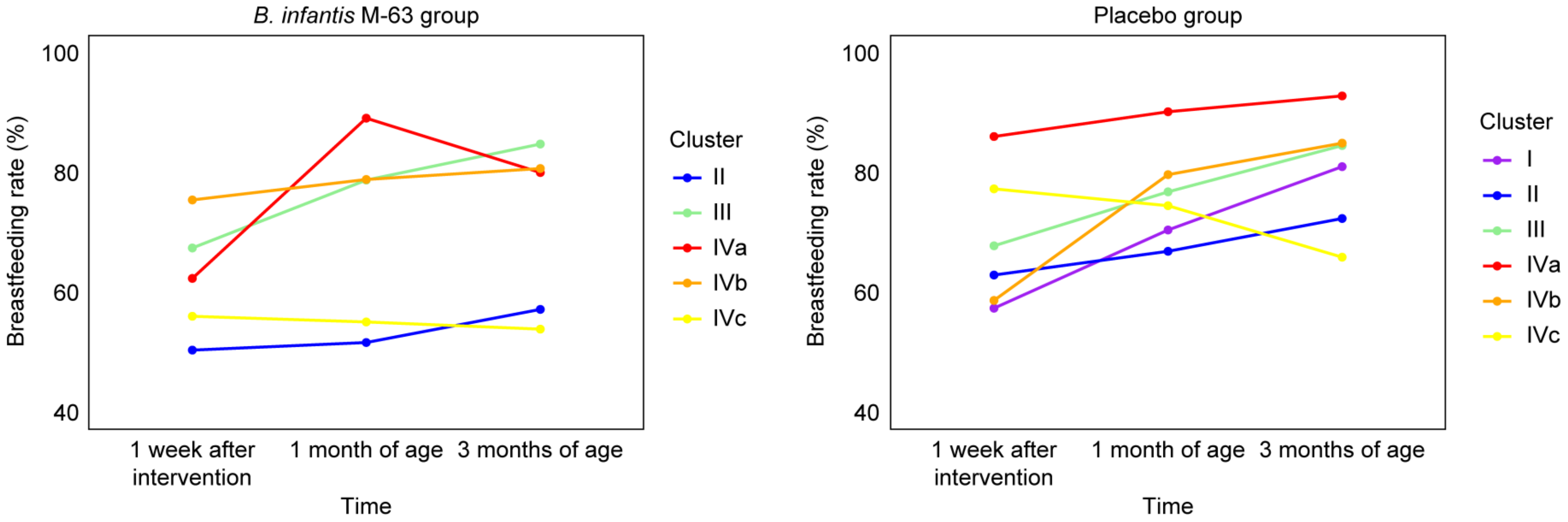

Figure S2. Changes in mean breastfeeding rates over time, as determined by cluster analysis for each group.
